# ^89^Zr-Labeled AR20.5: A MUC1-Targeting ImmunoPET Probe

**DOI:** 10.3390/molecules25102315

**Published:** 2020-05-15

**Authors:** Kimberly Fung, Delphine Vivier, Outi Keinänen, Elaheh Khozeimeh Sarbisheh, Eric W. Price, Brian M. Zeglis

**Affiliations:** 1 Department of Chemistry, Hunter College, City University of New York, New York, NY 10021, USA; kfung@gradcenter.cuny.edu (K.F.); dv402@hunter.cuny.edu (D.V.); omk2009@med.cornell.edu (O.K.); 2 Ph.D. Program in Chemistry, The Graduate Center of the City University of New York, New York, NY 10016, USA; 3 Department of Radiology, Memorial Sloan Kettering Cancer Center, New York, NY 10021, USA; 4 Department of Chemistry, University of Saskatchewan, Saskatoon, SK S7N 5B5, Canada; elaheh.khozeimeh@usask.ca (E.K.S.); eric.price@usask.ca (E.W.P.); 5 Department of Radiology, Weill Cornell Medical College, New York, NY 10021, USA

**Keywords:** mucin 1, MUC1, positron emission tomography, PET, AR20.5, zirconium-89

## Abstract

High expression levels of the tumor-associated antigen MUC1 have been correlated with tumor aggressiveness, poor response to therapy, and poor survival in several tumor types, including breast, pancreatic, and epithelial ovarian cancer. Herein, we report the synthesis, characterization, and in vivo evaluation of a novel radioimmunoconjugate for the immuno-positron emission tomography (immunoPET) imaging of MUC1 expression based on the AR20.5 antibody. To this end, we modified AR20.5 with the chelator desferrioxamine (DFO) and labeled it with the positron-emitting radiometal zirconium-89 (t_1/2_ ~3.3 d) to produce [^89^Zr]Zr-DFO-AR20.5. In subsequent in vivo experiments in athymic nude mice bearing subcutaneous MUC1-expressing ovarian cancer xenografts, [^89^Zr]Zr-DFO-AR20.5 clearly delineated tumor tissue, producing a tumoral activity concentration of 19.1 ± 6.4 percent injected dose per gram (%ID/g) at 120 h post-injection and a tumor-to-muscle activity concentration ratio of 42.4 ± 10.6 at the same time point. Additional PET imaging experiments in mice bearing orthotopic MUC1-expressing ovarian cancer xenografts likewise demonstrated that [^89^Zr]Zr-DFO-AR20.5 enables the visualization of tumor tissue—including metastatic lesions—with promising tumor-to-background contrast.

## 1. Introduction

Mucins are high molecular weight transmembrane glycoproteins that are expressed on the surface of normal and malignant epithelial cells and play a diverse collection of roles, ranging from the protection of the cell surface to the regulation of cellular adhesion [1,2,3]. MUC1 (also known as CA15.3 or polymorphic epithelial mucin) is a tumor-associated mucin that is over-expressed in most adenocarcinomas, including breast, pancreatic, and epithelial ovarian cancer [4,5,6,7,8,9]. The overexpression and aberrant expression of MUC1 have been linked to tumor aggressiveness and metastasis, poor response to therapy, and poor survival in several tumor types [10]. The role of MUC1 in ovarian cancer provides a representative case [11]. MUC1 is highly expressed in primary epithelial ovarian cancer as well as the vast majority of cases of metastatic disease [7,10]. To wit, patients with metastatic, treatment-resistant ovarian cancer frequently present elevated levels of MUC1, with >90% producing antibodies against the antigen [10,12,13]. Given its role in both transformation and metastatic progression, MUC1 has remained an enticing therapeutic target for almost three decades [10]. A wide variety of MUC1-targeted therapies have been developed over the years, including peptide-, protein-, and antibody-based vaccines, therapeutic antibodies, CAR-T cells, and alpha and beta particle-emitting radioimmunoconjugates [3,10,14,15,16,17,18,19,20,21,22,23]. Yet the field’s interest in MUC1-targeted therapeutics has ebbed and flowed during this period, as preclinical successes have proven difficult to recapitulate in the clinic. Recent years, however, have seen a reinvigoration of the area in response to the advent of checkpoint inhibitor therapies [24,25,26].

The last decade has played witness to a steady increase in the use of ^89^Zr-labeled antibodies as positron emission tomography (PET) imaging agents for the staging, treatment planning, and treatment monitoring of a range of cancers [27,28,29,30,31]. The surge in the popularity of ^89^Zr-immunoPET has been fueled by four factors: (1) the advent of monoclonal antibodies as clinical tools in the era of ‘personalized medicine’; (2) the advantageous match between the physical half-life of ^89^Zr (t_1/2_ ~3.3 d) and the in vivo residence time of antibodies; (3) the relatively facile production of the nuclide; and (4) the availability of an effective chelator for the radiometal (desferrioxamine, DFO). In light of this movement, the interest in MUC1-targeted therapies, and the role of MUC1 as a prognostic biomarker, we set out to create an immunoPET probe that could be used for the diagnostic and theranostic imaging of MUC1-expressing malignancies.

The monoclonal antibody that forms the foundation of our imaging agent is AR20.5, a murine IgG_1_ capable of binding MUC1 with high affinity and specificity [9]. While MUC1 is expressed by normal epithelial cells and cancer cells, the protein is aberrantly under-glycosylated in cancer cells [32]. This difference in glycosylation state between the epitopes of MUC1 expressed by healthy and malignant tissues has been exploited to create several antibodies capable of specifically binding the tumor-associated antigen, including AR20.5 [9]. To wit, AR20.5 binds in a glycosylation-dependent manner to the tandem repeat peptide sequence—i.e., DTRPAP—located within the variable number of tandem repeat (VNTR) region of the extracellular *N*-terminal domain of MUC1. Critically, this site is only accessible in tumor-associated epitopes of the mucin [33]. The therapeutic mechanism of AR20.5 is predicated on the antibody forming immune complexes with circulating MUC1 or MUC1-expressing tumor cells and subsequently inducing an immune response to the tumor itself [3]. A Phase I clinical trial employing AR20.5 as a monotherapy in 17 patients with MUC1-expressing cancers found that the antibody was generally well-tolerated and induced MUC1-specific immune responses but did not produce a significant therapeutic effect [3]. More recently, a therapeutic regimen of AR20.5 in combination with anti-programmed death-ligand (anti-PD-L1) and polyinosinic-polycytidylic acid (PolyICLC) was explored in mouse models of pancreatic cancer and was found to produce MUC1-specific immune responses that suppress tumor growth [34].

In the manuscript at hand, we report the synthesis, characterization, and in vivo evaluation of [^89^Zr]Zr-DFO-AR20.5 in two murine models of MUC1-expressing ovarian cancer. We have chosen ovarian cancer as the model system for this investigation because it is the fifth leading cause of cancer deaths amongst women—with ~14,000 deaths in the United States alone in 2019—and, as we have mentioned above, because the overexpression of MUC1 has been linked to aggressiveness, invasiveness, metastatic potential, and resistance to therapy in the disease [11,13,35]. Ultimately, we envision deploying [^89^Zr]Zr-DFO-AR20.5 for the immunoPET of all MUC1-expressing malignancies, both as a standalone diagnostic and prognostic tool and as a theranostic companion imaging agent for AR20.5 itself and other MUC1-targeted therapeutics. Finally, it is important to note that this investigation is admittedly not the first attempt to create a MUC1-targeted radiopharmaceutical for nuclear imaging. Indeed, several reports of MUC1-targeted PET and single photon emission computed tomography (SPECT) agents appear in the literature, including radioimmunoconjugates labeled with ^111^In, ^99m^Tc, and ^64^Cu, a pretargeted approach based on ^68^Ga, and—most recently—a ^89^Zr-labeled antibody [36,37,38,39,40,41]. However, several of these agents suffer from notable drawbacks, such as low activity concentrations in the tumor (e.g., [^64^Cu]Cu-DOTA-PR81), high activity concentrations in the kidneys (e.g., [^111^In]In-HMFG1-F(ab’)_2_), and high levels of complexity due to the need for genetic engineering (e.g., ‘Dock-and-Lock’ pretargeting with ^68^Ga) [36,37,38,39]. Taken together, we believe that the limitations of these earlier imaging strategies, the recent resurgence in MUC1-targeted therapeutics, and the potential of AR20.5 as a component of combination therapies create a strong rationale for the development of [^89^Zr]Zr-DFO-AR20.5.

## 2. Results

### 2.1. Modification of AR20.5 with DFO and Evaluation of the In Vitro Behavior of the Immunoconjugate in Human Ovarian Cancer Cells

**Figure 1:**
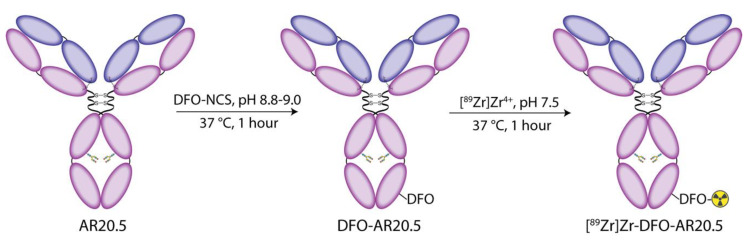
Schematic of the bioconjugation and radiosynthesis of [^89^Zr]Zr-DFO-AR20.5.

For the investigation at hand, AR20.5 was first modified with desferrioxamine (DFO), the ‘gold-standard’ chelator for the positron-emitting radiometal zirconium-89. To this end, a commercially available isothiocyanate-bearing derivative of DFO—*p*-SCN-Bn-DFO—was appended to the antibody via the ε-amines of solvent-exposed lysine residues according to published procedures (Figure 1) [42]. This approach to bioconjugation yields an immunoconjugate with an average of 1.2 ± 0.1 DFO/antibody as determined via matrix-assisted laser desorption/ionization (MALDI) mass spectrometry (Supplementary Figures S1 and S2 and Table S1). Subsequently, the in vitro behavior of DFO-AR20.5 was evaluated via flow cytometry experiments with MUC1-expressing SKOV3 human ovarian cancer cells, ultimately confirming the ability of the immunoconjugate to bind its molecular target (Supplementary Figure S3).

### 2.2. Radiolabeling of DFO-AR20.5 with Zr-89

After confirming the antigen-binding properties of DFO-AR20.5, the next step was to radiolabel the immunoconjugate with zirconium-89 (^89^Zr), a positron-emitting radiometal whose 3.3 d physical half-life aligns well with the multi-day circulation time of IgGs [27]. Standard published protocols were employed for the radiosynthesis, ultimately producing [^89^Zr]Zr-DFO-AR20.5 in >99% radiochemical purity, a radiochemical yield of 89 ± 10%, and a specific activity of 92.9 ± 10.0 MBq/mg [43]. Subsequently, a stability study in human serum at 37 °C revealed that 98.3 ± 0.2% of [^89^Zr]Zr-DFO-AR20.5 remains intact over an incubation period of 168 h (Supplementary Figure S4). 

### 2.3. Evaluation of the In Vivo Behavior of [^89^Zr]Zr-DFO-AR20.5 in Mice Bearing Subcutaneous SKOV3 Xenografts

**Figure 2:**
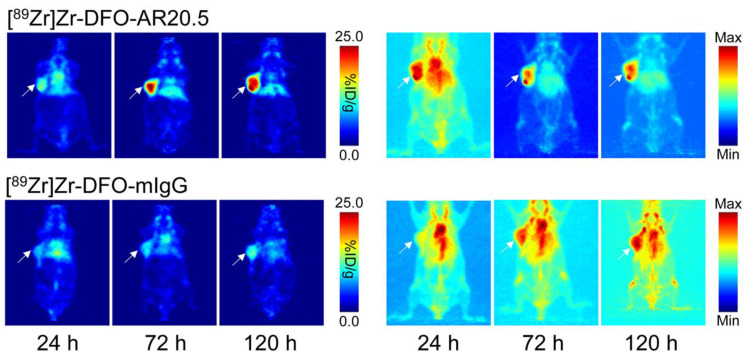
Planar (left) and maximum intensity projection (right; scaled to a minimum of 0% and a maximum of 100%) positron emission tomography (PET) images of representative athymic nude mice bearing subcutaneous SKOV3 xenografts collected at 24, 72, and 120 h following the intravenous tail vein injection of [^89^Zr]Zr-DFO-AR20.5 or [^89^Zr]Zr-DFO-mIgG. The white arrows mark the tumors.

To evaluate the in vivo performance of our new probe, small animal PET imaging and biodistribution studies were performed in athymic nude mice bearing subcutaneous, MUC1-expressing SKOV3 human ovarian cancer xenografts. PET images were obtained 24, 72, and 120 h after the intravenous administration of either [^89^Zr]Zr-DFO-AR20.5 (6.9–7.5 MBq, 93–102 μg) or an isotype control radioimmunoconjugate [^89^Zr]Zr-DFO-mIgG (6.9–7.1 MBq, 77–79 μg) via the tail vein. The PET images reveal that [^89^Zr]Zr-DFO-AR20.5 clearly delineates the MUC1-expressing tumor, with the tumoral activity concentrations growing over the course of the 120 h experiment (Figure 2, top). Critically, the minimal tumoral accumulation of the control radioimmunoconjugate—[^89^Zr]Zr-DFO-mIgG—illustrates that the uptake of [^89^Zr]Zr-DFO-AR20.5 in the tumor is antigen-mediated and not predicated on the enhanced permeability and retention effect alone (Figure 2, bottom). 

**Figure 3:**
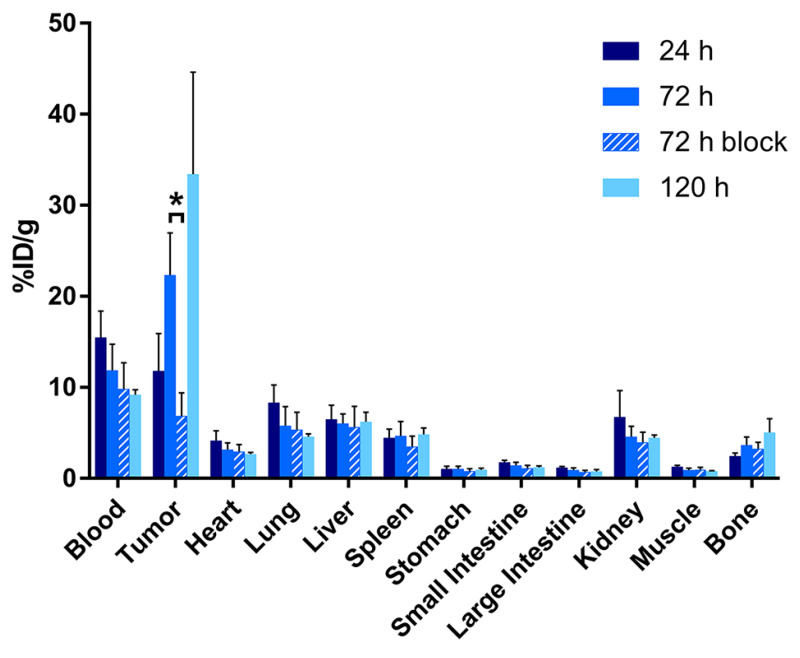
Biodistribution data from athymic nude mice (n = 5 per time point) bearing SKOV3 human ovarian cancer xenografts collected 24, 72, and 120 h after the intravenous administration of [^89^Zr]Zr-DFO-AR20.5 (0.65–0.69 MBq; 6.6–7.0 μg, in 200 μL 0.9% sterile saline). For the 72 h blocking experiment, the mice were administered the same dose of [^89^Zr]Zr-DFO-AR20.5 mixed with an excess of unmodified AR20.5 (~500 μg per mouse). * *p* = 0.0006.

The PET imaging results are reinforced by acute biodistribution data. To this end, mice (n = 5 per timepoint) were sacrificed at 24, 72, and 120 h after the administration of the ^89^Zr-labeled radioimmunoconjugates, and selected organs were harvested, washed, dried, and assayed on a gamma counter. These data confirm that the tumoral activity concentration of [^89^Zr]Zr-DFO-AR20.5 grows throughout the study, from 11.8 ± 4.1 percent injected dose per gram (%ID/g) at 24 h post-injection (p.i.) to 22.3 ± 4.6 %ID/g at 72 h p.i. to 33.4 ± 11.2 %ID/g at 120 h p.i (Figure 3 and Supplementary Table S2). A blocking experiment in which the tumor-bearing mice are administered a mixture of [^89^Zr]Zr-DFO-AR20.5 along with a vast excess of unlabeled AR20.5 (500 μg) demonstrates the specificity of the former: the tumoral activity concentration is 6.9 ± 2.5 %ID/g with blocking versus 22.3 ± 4.6 %ID/g without (*p* = 0.0006; Figure 3 and Supplementary Table S2). The biodistribution data also reveal that the background activity concentration of [^89^Zr]Zr-DFO-AR20.5 in the blood decreases from 15.5 ± 2.9 %ID/g at 24 h p.i. to 9.2 ± 0.6 %ID/g at 120 h p.i, as is typical for radioimmunoconjugates. In contrast, the activity concentration in the bone increases slightly over the course of the experiment (from 4.8 ± 1.8 %ID/g at 24 h p.i. to 6.6 ± 3.0 %ID/g at 120 h p.i.) in another phenomenon frequently observed with ^89^Zr-labeled antibodies. The activity concentrations in other healthy organs—including the liver, spleen, and kidneys—remain in the range of 2–7 %ID/g throughout the course of the experiment. Taken together, these biodistribution data yield tumor-to-healthy organ activity concentration ratios—e.g., tumor-to-blood, tumor-to-liver, and tumor-to-muscle activity concentration ratios of 3.6 ± 1.2, 5.4 ± 2.0, and 42.7 ± 14.6, respectively, at 120 h p.i.—that are generally favorable, though admittedly not extraordinary. 

### 2.4. Evaluation of the In Vivo Behavior of [^89^Zr]Zr-DFO-AR20.5 in Mice Bearing Orthotopic SKOV3-Red-FLuc Xenografts and Histopathological Analysis of Mouse Tumors and Metastases

**Figure 4:**
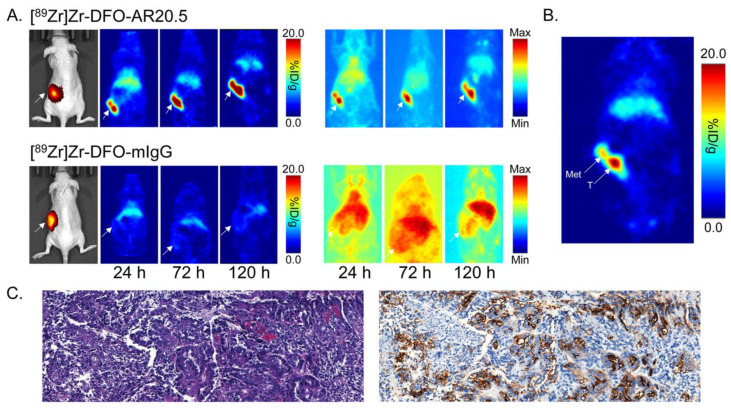
(**A**) Bioluminescence images (left) as well as planar (center) and maximum intensity projection (right; scaled to a minimum of 0% and maximum of 100%) PET images of representative athymic nude mice bearing orthotopic SKOV3-Red-FLuc xenografts obtained 24, 72, and 120 h following the intravenous tail vein injection of [^89^Zr]Zr-DFO-AR20.5 or [^89^Zr]Zr-DFO-mIgG. The white arrows mark the tumors; (**B**) Planar PET image of a representative athymic nude mouse bearing an orthotopic SKOV3-Red-FLuc xenograft collected at 120 h post-injection of [^89^Zr]Zr-DFO-AR20.5. The white arrows mark the tumor (T) and a peritoneal metastatic lesion (Met); (**C**) Hematoxylin and eosin staining (10× magnified; left) and immunohistochemical staining (10× magnified; right) of the peritoneal metastatic lesion from the representative mouse, with brown staining indicating the expression of MUC1.

With the subcutaneous xenograft data in hand, the next step was to evaluate [^89^Zr]Zr-DFO-AR20.5 in a more realistic orthotopic xenograft model. To this end, orthotopic human ovarian cancer xenografts were established in athymic nude mice via the injection of MUC1- and luciferase-expressing SKOV3-Red-FLuc cells into the fat pad surrounding the ovary. Subsequent PET imaging experiments revealed that the xenografts in the left ovary can be clearly delineated as early as 24 h post-injection, with the activity concentration continuing to rise throughout the experiment (Figure 4). As in the experiments with the subcutaneous xenograft model, PET imaging using an isotype control radioimmunoconjugate—[^89^Zr]Zr-DFO-mIgG—produced little tumoral accumulation, reinforcing the specificity of the MUC1-targeting imaging agent. 

After the final imaging time point, the orthotopic tumor-bearing mice were sacrificed, and selected tissues were harvested, washed, weighed, and assayed for ^89^Zr using a gamma counter to produce quantitative biodistribution data. Not surprisingly, these data are consistent with the imaging results, pointing to a tumoral activity concentration of 11.3 ± 7.1 %ID/g at 120 p.i. but also significant accumulation in the liver (10.5 ± 2.4 %ID/g) and spleen (6.1 ± 0.3 %ID/g) (Supplementary Table S4). The latter is most likely the result of the formation of immune complexes between shed MUC1 and circulating radioimmunoconjugate that were then deposited in these tissues. Interestingly, focal uptake of [^89^Zr]Zr-DFO-AR20.5 was also observed in several lesions that appeared to be metastases in the abdomen, peritoneum, liver, and right ovary of the orthotopic tumor-bearing mice (Supplementary Figure S5). Histopathological analysis revealed that these lesions were composed of neoplastic cells arranged in solid nests and scattered tubules, confirming them as ovarian cell carcinoma (Figure 4). Furthermore, immunohistochemical staining revealed that these metastatic lesions do, indeed, express MUC1 (Figure 4).

## 3. Discussion

In the preceding pages, we have described the synthesis, characterization, and in vivo evaluation of [^89^Zr]Zr-DFO-AR20.5, a radioimmunoconjugate for the PET imaging of MUC1-expressing tumors. The chemical and biological characterization experiments clearly demonstrate that the bioconjugation of AR20.5 does not adversely affect the ability of the antibody to bind its target antigen. Moving on to the in vivo experiments, the data show that [^89^Zr]Zr-DFO-AR20.5 is capable of clearly visualizing MUC1-expressing tumor tissue in mice bearing both subcutaneous and orthotopic ovarian cancer xenografts. With respect to the former, the in vivo behavior of [^89^Zr]Zr-DFO-AR20.5 in mice bearing subcutaneous MUC1-positive ovarian cancer xenografts mirrors that of other ^89^Zr-labeled radioimmunoconjugates. To wit, while the activity concentration in tumor tissue increases over the course of the experiment, that in the blood and several other well-perfused organs (e.g., the lungs) decreases in kind. A small amount of radioactivity was observed in the bones of the mice, likely the result of the in vivo demetallation of the radioimmunoconjugate and the subsequent deposition of osteophilic [^89^Zr]Zr^4+^ in the bone. However, these values are consistent with those observed in preclinical imaging and biodistribution experiments using other ^89^Zr-labeled antibodies. 

While the tumor-to-muscle activity concentration ratios in these mice were quite high (42.7 ± 14.6 at 120 h p.i.), several key tumor-to-healthy organ activity concentration ratios—most notably tumor-to-blood (3.6 ± 1.2 at 120 h p.i.), tumor-to-liver (5.4 ± 2.0 at 120 h p.i.), and tumor-to-spleen (6.9 ± 2.5 at 120 h p.i.)—lie below the impressive ‘double digit’ values observed for several other ^89^Zr-labeled radioimmunoconjugates, such as [^89^Zr]Zr-DFO-J591, [^89^Zr]Zr-DFO-trastuzumab, and [^89^Zr]Zr-DFO-pertuzumab. This phenomenon is most likely related to the shedding of MUC1 from the tumor cells and the formation of macromolecular MUC1-[^89^Zr]Zr-DFO-AR20.5 immune complexes that can persist in the blood and subsequently accumulate in the liver and spleen. However, our previous preclinical data with the CA125-targeted radioimmunoconjugate [^89^Zr]Zr-DFO-B43.13 as well as the preclinical and clinical performance of the CA19.9-targeted radioimmunoconjugate [^89^Zr]Zr-DFO-5B1 demonstrate the feasibility of the immunoPET imaging of shed antigens [44,45,46]. Furthermore, the in vivo performance of [^89^Zr]Zr-DFO-AR20.5 compares favorably to that of two recently reported MUC1-targeted radioimmunoconjugates: [^64^Cu]Cu-DOTA-PR81 and [^89^Zr]Zr-DFO-GGSK-1/30 [36,41]. While direct comparisons are dubious due to the use of different tumor models, the tumoral activity concentrations and tumor-to-healthy-organ activity concentration ratios of [^89^Zr]Zr-DFO-AR20.5 exceed those created by the former and are roughly equivalent to those produced by the latter. Finally, the in vivo data in mice bearing orthotopic xenografts largely reflects the data collected in the subcutaneous tumor-bearing animals. Yet the ability of [^89^Zr]Zr-DFO-AR20.5 to visualize several abdominal, peritoneal, hepatic, and ovarian metastatic lesions in these mice further underscores its potential as a tool for the non-invasive staging of the disease.

Moving forward, we plan to continue to explore the use of [^89^Zr]Zr-DFO-AR20.5 for both diagnostic and theranostic imaging in ovarian cancer. Along these lines, we are currently formulating plans to explore the in vivo performance of [^89^Zr]Zr-DFO-AR20.5 in mice bearing patient-derived ovarian cancer xenografts. Furthermore, we plan to interrogate the utility of [^89^Zr]Zr-DFO-AR20.5 as a predictive theranostic imaging agent in human MUC1 transgenic mice (MUC.Tg) undergoing AR20.5/anti-PD-L1/PolyICLC combination therapy. Finally, we have also begun exploring whether the in vivo performance of [^89^Zr]Zr-DFO-AR20.5 could benefit from the removal of its heavy chain glycans—and thus the abrogation of its interactions with murine FcγRI receptors—a phenomenon that we have previously studied [47,48]. In this case, we believe that the modulation of the interactions between [^89^Zr]Zr-DFO-AR20.5 and the murine immune system may be particularly important given the therapeutic mechanism of the antibody. In the end, this pilot investigation has clearly demonstrated that [^89^Zr]Zr-DFO-AR20.5 is an effective tool for the non-invasive delineation of MUC1-expressing tumor tissue, and we are hopeful that in the future, this radioimmunoconjugate can play an important role in the clinical diagnostic and theranostic imaging of ovarian cancer.

## 4. Materials and Methods 

Unless otherwise noted, all chemicals were purchased from Sigma-Aldrich (St. Louis, MO, USA) or Fisher Scientific (Pittsburgh, PA, USA) and were used without further purification. All water used was ultrapure (> 18.2 MΩcm^−1^ at 25 °C), and dimethylsulfoxide was of molecular biology grade (> 99.9%). *p*-SCN-Bn-DFO was purchased from Macrocyclics, Inc. (Plano, TX, USA). MALDI mass spectrometry was performed according to previously reported methods by the Alberta Proteomics and Mass Spectrometry Facility (University of Alberta, Edmonton, AB, Canada). ^89^Zr was produced and purified via the ^89^Y(*p,n*)^89^Zr reaction at Memorial Sloan Kettering Cancer Center as [^89^Zr]Zr-oxalate in 1.0 M oxalic acid. The AR20.5 antibody was obtained from Quest Pharmatech, Inc. (Edmonton, AB, Canada), and mouse IgG_1_ (mIgG, #31903) was purchased from ThermoFisher Scientific (Waltham, MA, USA). All in vivo experiments were performed in accordance with published protocols approved by the Institutional Animal Care and Use Committees of Hunter College (5/18-02), Weill Cornell Medical College (2015-004), and Memorial Sloan Kettering Cancer Center (A3311-01).

### 4.1. Instrumentation

All instruments were calibrated and maintained according to standard quality control practices and procedures. UV-Vis measurements were taken on a Shimadzu BioSpecNano Micro-volume UV-Vis Spectrophotometer (Shimadzu Scientific Instruments, Kyoto, Japan). Radioactivity measurements were taken using a CRC-15R Dose Calibrator (Capintec, Inc., Ramsey, NJ, USA), and biodistribution samples were counted on a calibrated Automatic Wizard^2^ γ-counter (PerkinElmer, Inc., Waltham, MA, USA). The radiolabeling of the immunoconjugate was monitored using glass-fiber, silica-impregnated instant thin-layer chromatography (iTLC) paper (Pall Corp., East Hills, NY, USA) and analyzed on an AR-2000 radio-TLC plate reader using Winscan Radio-TLC software (Bioscan, Inc., Washington, DC, USA).

### 4.2. Modification of Antibodies with DFO

AR20.5 (800 µg, 5.4 nmol) was dissolved in 500 µL of Chelex-treated (Chelex^®^ 100 Resin, Bio-Rad Laboratories, Inc., Hercules, CA, USA) phosphate-buffered saline (Chelex PBS, pH 7.4), and the pH of the solution was adjusted to 8.8–9.0 with Na_2_CO_3_ (0.1 M). 5 equivalents of *p*-SCN-Bn-DFO (13.5 µL, 1.5 mg/mL in DMSO) were added to the solution in small aliquots. The resulting solution was incubated at 37 °C for 1 h with shaking at 500 rpm. The DFO-modified antibody was then purified using size exclusion chromatography (Sephadex G-25 M, PD-10 column, GE Healthcare, Chicago, IL, USA; dead volume: 2.5 mL, eluted with 2 mL of Chelex PBS, pH 7.4) and concentrated using centrifugal filtration units with a 50,000 Da molecular weight cut-off (Amicon^TM^ Ultra 2 mL Centrifugal Filtration Units, Millipore, Sigma Corp., Burlington, MA, USA). DFO-mIgG was prepared as previously described, with the following modifications: 500 µg (3.3 nmol) of mIgG and 8.4 µL of *p*-SCN-Bn-DFO (1.5 mg/mL in DMSO) were used.

#### Degree of Labeling Determination via MALDI-ToF Mass Spectrometry

Matrix-assisted laser desorption/ionization (MALDI) mass spectrometry was used to determine the number of DFO moieties per antibody (Alberta Proteomics and Mass Spectrometry Facility, University of Alberta, Canada). The immunoconjugates were analyzed in triplicate using a Bruker Ultraflex MALDI-ToF/ToF (Bruker Daltonik GmbH, Bremen, Germany). To this end, 1 µL of each sample (1 mg/mL) was mixed with 1 µL of sinapic acid (10 mg/mL in 50% acetonitrile/water and 0.1% trifluoroacetic acid). 1 µL of the sample/matrix solution was then spotted onto a stainless-steel target plate and allowed to air dry. Ions were analyzed in positive mode, and external calibration was performed using a standard protein mixture (bovine serum albumin). The difference between the mass of each DFO-bearing immunoconjugate and its unmodified parent antibody (Supplementary Table S1) was calculated, and the degree of labeling was determined via division by the mass of *p*-SCN-Bn-DFO. 

### 4.3. Radiolabeling with ^89^Zr

590 µg of DFO-AR20.5 were diluted in 400 µL of Chelex PBS, pH 7.4. [^89^Zr]Zr-oxalate (1640 µCi) in 1.0 M oxalic acid was adjusted to pH 7.0–7.5 with 1.0 M Na_2_CO_3_, resulting in a total volume of 95 µL. After the bubbling of CO_2_ ceased, the ^89^Zr solution was added to the antibody solution, and the resulting mixture was placed on an agitating thermomixer at 500 rpm for 1 h at 25 °C. The progress of the reaction was then assayed using radio-TLC with an eluent of 50 mM EDTA (pH 5.0). Subsequently, the reaction was quenched with 10 µL of 50 mM EDTA (pH 5.0), and the immunoconjugate was purified using size exclusion chromatography (Sephadex G-25 M, PD-10 column, GE Healthcare; dead volume: 2.5 mL, eluted with 500 µL fractions of Chelex PBS, pH 7.4) and concentrated using centrifugal filtration units with a 50,000 Da molecular weight cut-off (Amicon^TM^ Ultra 2 mL Centrifugal Filtration Units, Millipore, Sigma Corp., Burlington, MA, USA). The radiochemical purity of the final radiolabeled construct was assayed via radio-TLC using 50 mM EDTA (pH 5.0) as an eluent. In the radio-TLC experiments, the radioimmunoconjugate remains at the baseline, while free [^89^Zr]Zr^4+^ cations and [^89^Zr]Zr-EDTA travel with the solvent front. For the radiolabeling of DFO-mIgG, 340 µg of the construct and 950 µCi of [^89^Zr]Zr-oxalate (total volume of 50 µL) were used. All radiolabeling studies were performed in triplicate. 

### 4.4. Cell Culture

Human ovarian cancer cell line SKOV3 was purchased from the American Type Culture Collection (ATCC, Manassas, VA, USA) and maintained in McCoy’s 5A Medium supplemented with 10% heat-inactivated fetal calf serum, 100 units/mL penicillin, and 100 units/mL streptomycin in an incubator (Heracell^TM^ 150i, ThermoFisher Scientific) set to 37 °C and 5% CO_2_. SKOV3-Red-FLuc—a human ovarian cancer cell line that expresses a red-shifted firefly luciferase gene that enables bioluminescence imaging—was purchased from PerkinElmer, Inc. (Waltham, MA, USA) and maintained in McCoy’s 5A Medium supplemented with 10% heat-inactivated fetal calf serum. The cell lines were harvested and passaged upon reaching 80% confluency using 0.25% trypsin/0.53 mM EDTA in Hank’s Buffered Salt Solution without calcium and magnesium. All media was purchased from the Media Preparation Facility at Memorial Sloan Kettering Cancer Center.

### 4.5. Flow Cytometry

The MUC1-expressing human ovarian cancer cell line SKOV3 was used for flow cytometry experiments. 50 µL of DFO-AR20.5 at 24, 12, 6, 3, 1.5, 0.75, 0.375, and 0.1875 µg/µL was incubated with 1 × 10^6^ cells/mL for 30 min on ice. The cells were washed with 1 mL of ice-cold PBS three times by pelleting and resuspension. The cells were then incubated with 50 µL of a goat anti-mouse IgG-AlexaFluor568 secondary antibody (ThermoFisher Scientific) at 8 µg/µL for 30 min on ice. Subsequently, the cells were again washed with ice-cold PBS and then analyzed on a BD LSR II flow cytometer (BD Biosciences, San Jose, CA, USA). Binding data was collected in triplicate for each experimental condition and then plotted using FlowJo^TM^ software (BD, Franklin Lakes, NJ, USA). 

### 4.6. Radioimmunoconjugate Stability Assays

The stability of the radioimmunoconjugate with respect to the loss of radioactivity was investigated by incubating [^89^Zr]Zr-DFO-AR20.5 in human serum for 7 days at 37 °C with shaking at 500 rpm (n = 3). At predetermined times, the radiochemical purity of [^89^Zr]Zr-DFO-AR20.5 was determined in triplicate via radio-TLC with an eluent of 50 mM EDTA (pH 5.0).

### 4.7. Xenograft Models

Six to eight-week-old female athymic nude mice were obtained from either Charles River Laboratories (Wilmington, MA, USA) or The Jackson Laboratory (Bar Harbor, ME, USA) and allowed to acclimatize for approximately 1 week prior to inoculation. Animals were housed in ventilated cages and given water and food ad libitum.

#### 4.7.1. Subcutaneous Xenograft Model

Mice were anaesthetized by inhalation of 2% isoflurane (Baxter Healthcare, Deerfield, IL, USA)/oxygen gas mixture and xenografted subcutaneously on the left shoulder with 5 × 10^6^ SKOV3 cells in a 150 µL cell suspension of a 1:1 mixture of fresh media: Matrigel (Corning Life Sciences, Corning, NY, USA). The SKOV3 tumors reached the ideal size for imaging and biodistribution studies (~100–150 cm^3^) after approximately 6 weeks.

#### 4.7.2. Orthotopic Xenograft Model

Mice were anaesthetized by inhalation of 1.5% isoflurane/oxygen gas mixture, and surgery was performed on a heated surface to maintain body temperature. One dose of meloxicam (2 mg/kg) and buprenorphine (0.5 mg/kg) was given preemptively via subcutaneous injection. Bupivacaine, a local anesthetic agent, was injected into the tissue adjacent to the incision line. The skin was then prepped for surgery by alternating scrubs of povidone-iodine and 70% ethanol. A dorsolateral incision (1–2 cm in length) was made on the skin on the top right of the spleen, and the retroperitoneum was dissected to expose the fat pad surrounding the ovary. Then, 8 × 10^5^ SKOV3-Red-FLuc cells in 15 µL of PBS were injected into the left ovary. The retroperitoneum was closed using Vicryl sutures. The skin was closed with sterile wound clips, and the wound edges were sealed with a few drops of tissue adhesive. The tumors reached the ideal size for experiments after approximately 10 weeks.

To monitor tumor growth, mice were monitored using bioluminescence imaging at 4 and 8 weeks. Approximately 5 min prior to bioluminescence imaging, mice were anesthetized by inhalation of 2% isoflurane/oxygen gas mixture, administered 150 µL luciferin (4.5 mg per mouse; 30 mg/mL; RediJect d-Luciferin Bioluminescent Substrate, PerkinElmer, Inc.) via intraperitoneal injection, and placed on the scanner bed. Anesthesia was maintained by inhalation of 1% isoflurane/oxygen gas mixture. An exposure time of 0.5 s, binning factor of 8, f/stop of 1, and field of view of 13.4 cm were used. The resulting images were processed using Living imaging (v4.4) software.

### 4.8. PET Imaging

PET imaging was conducted on a microPET Focus 120 small-animal scanner (Siemens Medical Solutions, Malvern, PA, USA). Approximately 5 min prior to PET image acquisition, mice were anaesthetized by inhalation of 2% isoflurane/oxygen gas mixture and kept under anesthesia for the duration of the scan. Static scans were recorded 24, 72, and 120 h after the intravenous tail vein administration of either [^89^Zr]Zr-DFO-AR20.5 (6.9–7.5 MBq, 93–102 μg, in 200 μL 0.9% sterile saline) or [^89^Zr]Zr-DFO-mIgG (6.9–7.1 MBq, 77–79 μg, in 200 μL 0.9% sterile saline) for a total scan time of 10 min. An energy window of 350–700 keV and a coincidence timing window of 6 nanoseconds were used. Data were sorted into 2-dimensional histograms by Fourier re-binning, and transverse images were reconstructed by filtered back-projection (FBP). The imaging data were then normalized to correct for the non-uniformity of response of the detector, physical decay of the radionuclide to the time of injection, dead-time count losses, and positron-branching ratio, but no attenuation, scatter, or partial-volume averaging correction was applied. The counting rates in the reconstructed images were converted to activity concentrations (percentage injected dose per gram of tissue [%ID/g]) using a system calibration factor derived from the imaging of a mouse-sized water-equivalent phantom containing ^89^Zr. Maximum intensity projection (MIP) images were generated from 3-dimensional ordered subset expectation maximization reconstruction (3D-OSEM). The resulting images were analyzed using ASIPro VM^TM^ software (Concorde Microsystems, Knoxville, TN, USA). Cohorts of 4 were used for the mice bearing subcutaneous xenografts, while a cohort of 3 was used for the mice bearing orthotopic xenografts (due to the low take-rate of the orthotopic tumors). 

### 4.9. Acute Biodistribution

Athymic nude mice bearing subcutaneous SKOV3 (left shoulder, 100–150 mm^3^, n = 5 per cohort) were randomized prior to the study and were warmed gently with a heat lamp for 5 min prior to the administration of [^89^Zr]Zr-DFO-AR20.5 (0.65–0.69 MBq; 6.6–7.0 μg, in 200 μL 0.9% sterile saline) via tail vein injection (t = 0). For the 72 h blocking experiment, the mice were administered the same dose of [^89^Zr]Zr-DFO-AR20.5 mixed with an excess of unmodified AR20.5 (~500 μg per mouse). The mice were euthanized via CO_2_ (g) asphyxiation at 24, 72, and 120 h post-injection, and 13 tissues (including tumor) were collected, rinsed in water, dried, weighed, and counted using a gamma counter calibrated for ^89^Zr. Counts were converted into activity units (µCi) using a calibration curve generated from known standards. The number of counts per minute in each tissue was background and decay corrected to the injection time. The %ID/g for each sample was calculated by normalization to the total injected activity. 

The acute biodistribution studies with the mice bearing orthotopic SKOV3-Red-FLuc xenografts (n = 3 per cohort) were performed in a similar manner. In this case, however, the same mice were used for the PET imaging and acute biodistribution experiments (see Methods and Materials Section 4.8). After the final imaging time point (i.e., 120 h p.i.), the cohorts of mice were euthanized, and the biodistribution study was performed as described above. 

### 4.10. Histopathology

The tumors and metastases harvested for the biodistribution were fixed in 10% neutral buffered formalin and stored until the radioactivity decayed for 10 half-lives. The formalin-fixed tissue samples were processed in ethanol and xylene, embedded in paraffin, sectioned into 5 µm thick sections, and stained with hematoxylin and eosin (H&E). Sections from the tumors and metastases were also stained by immunohistochemistry for MUC1 on a Leica Bond RX automated staining platform (Leica Biosystems, Wetzlar, Germany). Subsequently, heat-induced epitope retrieval was conducted in a pH 9.0 buffer, and the primary antibody (MUC1 antibody, ThermoFisher Scientific, MA532265) was applied using a 1:250 dilution. Lastly, a polymer detection system (Novocastra Bond Polymer Refine Detection, Leica Biosystems, DS9800) was applied. The resulting slides were interpreted by a board-certified veterinary pathologist from the Laboratory of Comparative Pathology at Memorial Sloan Kettering Cancer Center. The slides were digitized using Pannoramic Flash scanners (3DHistech). The scanned images were processed using Pannoramic Viewer software (3DHistech).

### 4.11. Statistical Analysis

Results of the assays presented are expressed as the mean ± standard deviation of three independent experiments. Statistical differences were analyzed with GraphPad Prism software (7.0 GraphPad Software Inc., San Diego, CA, USA) via an unpaired, two-tailed Student’s t test (with a Welch’s correction). 

## Supplementary Materials

The following are available online: Figure S1: Representative MALDI-ToF spectrum for AR20.5; Figure S2: Representative MALDI-ToF spectrum for DFO-AR20.5; Figure S3: Flow cytometry analysis of DFO-AR20.5 with SKOV3 human ovarian cancer cells; Figure S4: Stability of [^89^Zr]Zr-DFO-AR20.5 in human serum at 37 °C; Figure S5: Biodistribution data and PET images for athymic nude mice bearing orthotopic SKOV3-Red-FLuc xenografts; Table S1: Degree of labeling of DFO-AR20.5 as determined via MALDI-MS; Table S2: Biodistribution data from athymic nude mice bearing subcutaneous SKOV3 human ovarian cancer xenografts; Table S3: Tumor-to-background activity concentration ratios calculated using the biodistribution data from athymic nude mice bearing subcutaneous SKOV3 human ovarian cancer xenografts; Table S4: Biodistribution data from athymic nude mice bearing orthotopic SKOV3-Red-FLuc human ovarian cancer xenografts; Table S5: Tumor-to-background activity concentration ratios derived from the biodistribution data from athymic nude mice bearing orthotopic SKOV3-Red-FLuc human ovarian cancer xenografts; Table S6: Biodistribution data for the metastatic lesions collected from athymic nude mice bearing orthotopic SKOV3-Red-FLuc human ovarian cancer xenografts.
