# Supplementary material for: 89Zr-Labeled AR20.5: A MUC1-Targeting ImmunoPET Probe
Source: Molecules. 2020 May 15;25(10):2315. doi: 10.3390/molecules25102315 (PMC7287814; doi:10.3390/molecules25102315)
Supplement: Supplementary file 1 [file molecules-25-02315-s001.pdf]

Supplementary Materials

^{89}Zr-Labeled AR20.5: A MUC1-Targeting ImmunoPET Probe

Kimberly Fung[1,2,†], Delphine Vivier[1,†], Outi Keinänen[1,3], Elaheh Khozeimeh Sarbisheh[4], Eric W. Price[4], Brian M. Zeglis[1,2,3,5,*]

[1] Department of Chemistry, Hunter College, City University of New York, New York 10021, NY, U.S.A.

[2] Ph.D. Program in Chemistry, The Graduate Center of the City University of New York, New York, NY 10016, U.S.A.

[3] Department of Radiology, Memorial Sloan Kettering Cancer Center, New York, NY 10021, U.S.A.

[4] Department of Chemistry, University of Saskatchewan, Saskatoon SK S7N 5B5, Canada

[5] Department of Radiology, Weill Cornell Medical College, New York, NY 10021, U.S.A.

***** Correspondence: bz102@hunter.cuny.edu; Tel.: +1-212-896-0443; Fax: +1-212-772-5332

[†] These two authors contributed equally to this work

Supplemental Figures

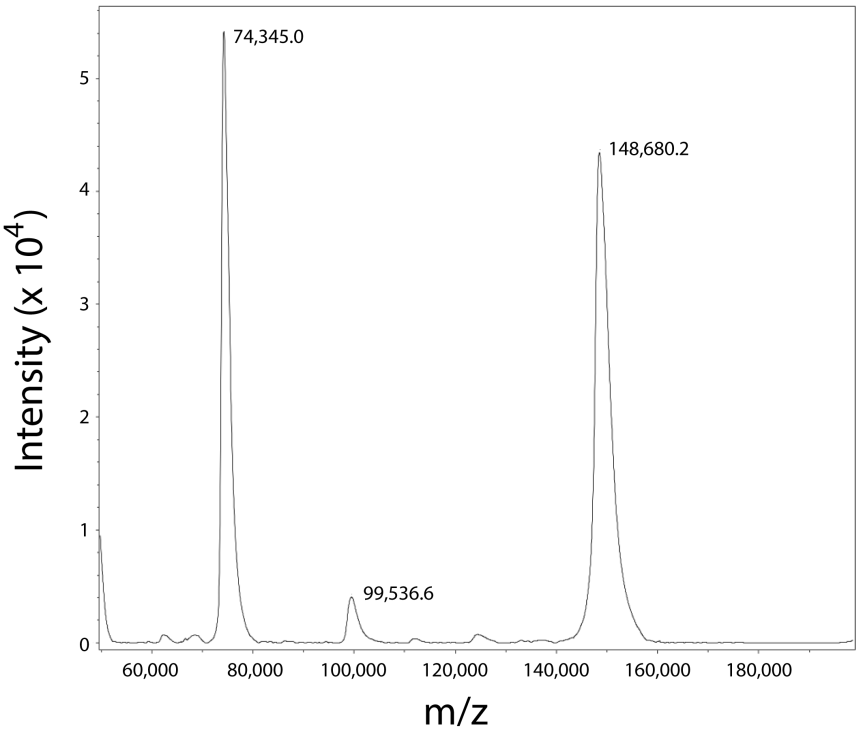

Figure S1. Representative MALDI-ToF spectrum for AR20.5.

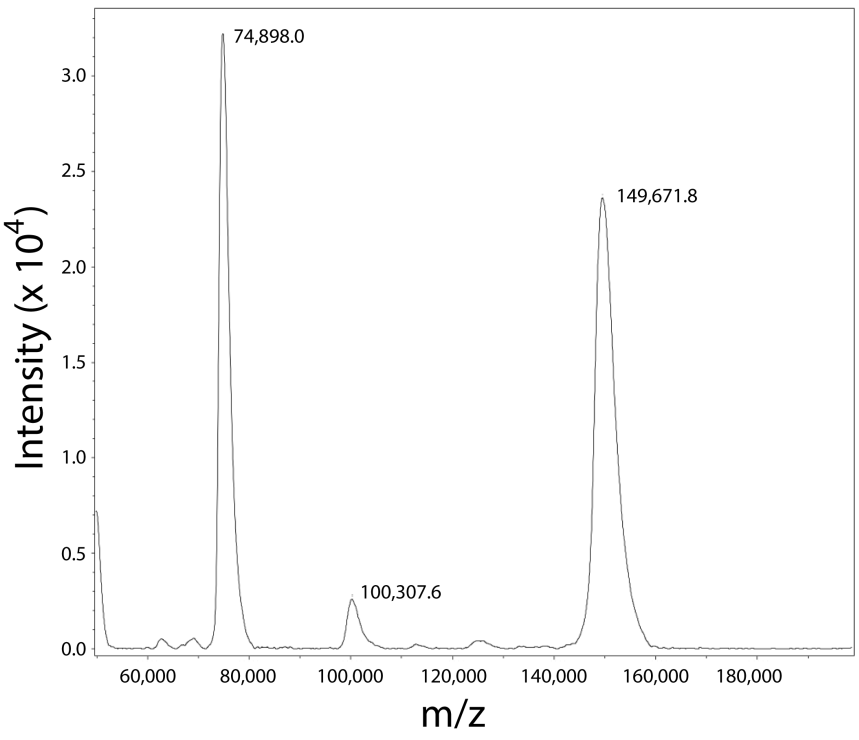

Figure S2. Representative MALDI-ToF spectrum for DFO-AR20.5.

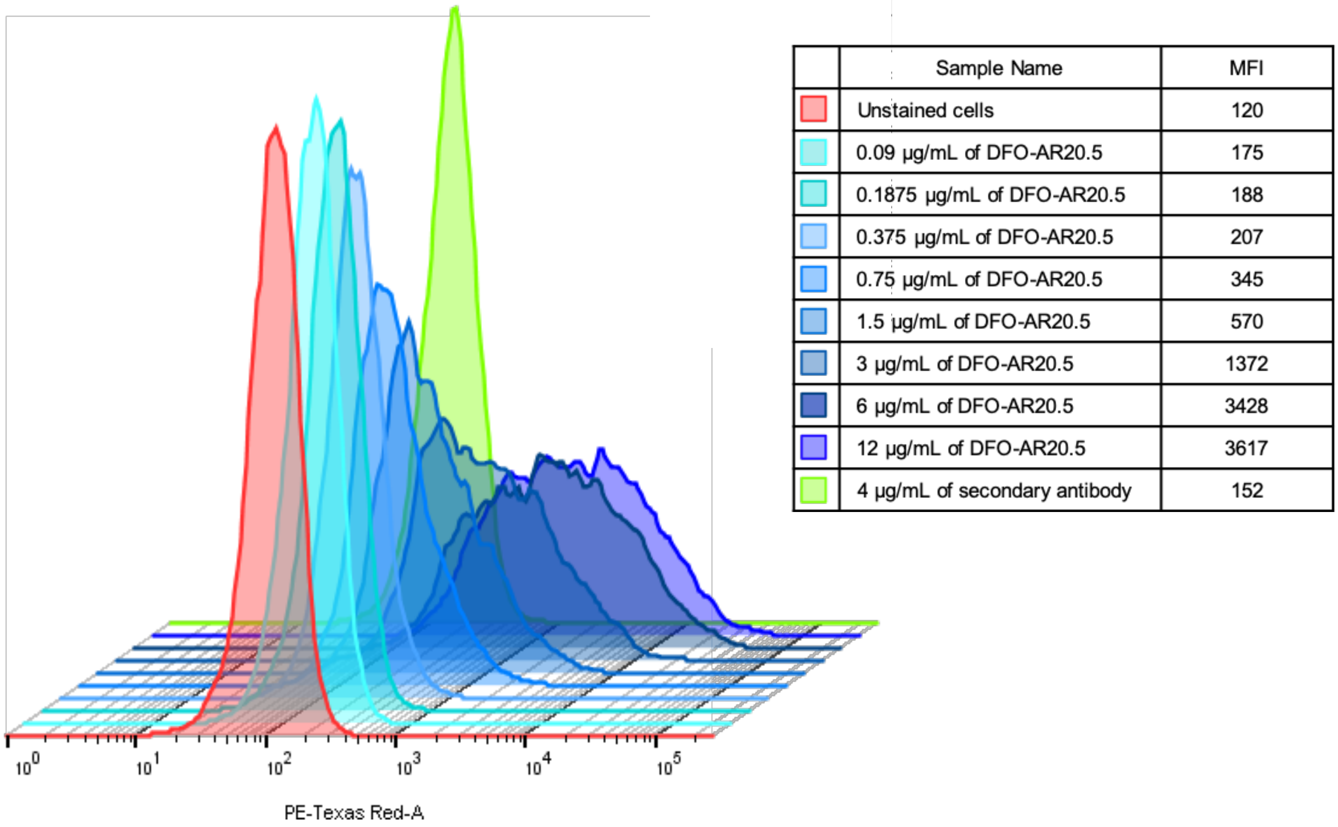

	Sample Name	MFI
	Unstained cells	120
	0.09 µg/mL of DFO-AR20.5	175
	0.1875 µg/mL of DFO-AR20.5	188
	0.375 µg/mL of DFO-AR20.5	207
	0.75 µg/mL of DFO-AR20.5	345
	1.5 µg/mL of DFO-AR20.5	570
	3 µg/mL of DFO-AR20.5	1372
	6 µg/mL of DFO-AR20.5	3428
	12 µg/mL of DFO-AR20.5	3617
	4 µg/mL of secondary antibody	152

Figure S3. Flow cytometry analysis of DFO-AR20.5 with SKOV3 human ovarian cancer cells. MFI = mean fluorescence intensity.

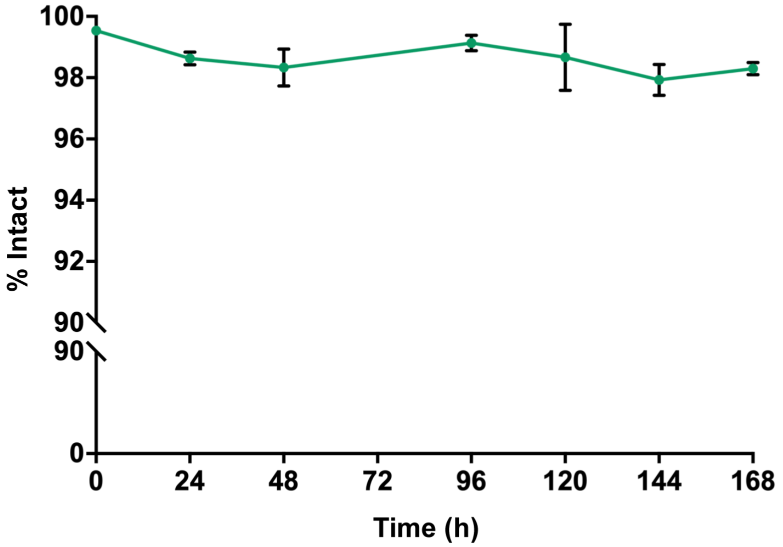

Figure S4. The stability of [^{89}Zr]Zr-DFO-AR20.5 in human serum at 37 °C. Measurements were collected using radioTLC and were performed in triplicate.

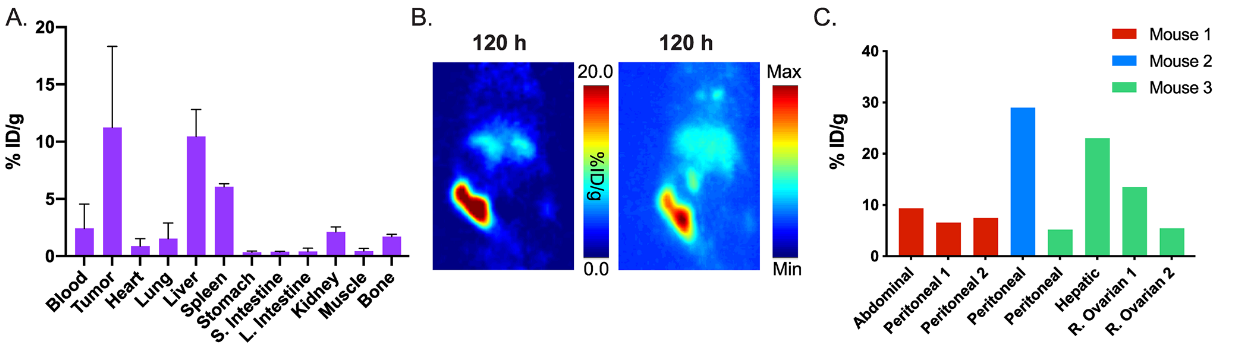

Figure S5. (A) Biodistribution data for athymic nude mice (n = 3) bearing orthotopic SKOV3-Red-FLuc xenografts collected 120 h following the intravenous tail vein injection of [^{89}Zr]Zr-DFO-AR20.5 (5.6 – 6.0 MBq, 150.6 – 161.4 μCi, 59.1 – 63.3 μg; in 200 μL 0.9% sterile saline); (B) Planar (left) and maximum intensity projection (MIP, right; scaled to a minimum of 0% and a maximum of 100%) PET images of a representative athymic nude mouse — "Mouse 2" — bearing an orthotopic SKOV3-Red-FLuc xenograft collected 120 h post-injection of [^{89}Zr]Zr-DFO-AR20.5; (C) Biodistribution data for the metastatic lesions collected from the orthotopic tumor-bearing mice.

Supplemental Tables

Table S1. Degree of labeling of DFO-AR20.5 (n = 3) as determined via MALDI-ToF mass spectrometry

Immunoconjugate	Average mass (Da)	Degree of Labeling (DFO/mAb)
AR20.5	148716 ± 49	N/A
DFO-AR20.5	149652 ± 53	1.2 ± 0.1

Table S2. Biodistribution data from athymic nude mice (n = 5 per time point) bearing SKOV3 human ovarian cancer xenografts collected 24, 72, and 120 h after the intravenous administration of [^{89}Zr]Zr-DFO-AR20.5 (0.65 – 0.69 MBq; 17.6 – 18.6 µCi; 6.6 – 7.0 µg). For the 72 h blocking experiment, the mice were administered the same amount of [^{89}Zr]Zr-DFO-AR20.5 mixed with an excess of unmodified AR20.5 (~500 µg per mouse). The values are %ID/g ± SD. Stomach, small intestine, and large intestine values include contents.

**Tissue**	**24 h**	**72 h**	**72 h block**	**120 h**
Blood	15.5 ± 2.9	11.9 ± 2.9	9.8 ± 2.9	9.2 ± 0.6
Tumor	11.8 ± 4.1	22.3 ± 4.6	6.9 ± 2.5	33.4 ± 11.2
Heart	4.2 ± 1.1	3.2 ± 0.7	3.0 ± 0.8	2.7 ± 0.2
Lungs	8.3 ± 1.9	5.8 ± 2.1	5.3 ± 1.9	4.6 ± 0.3
Liver	6.5 ± 1.6	6.0 ± 1.1	5.6 ± 2.3	6.2 ± 1.1
Spleen	4.5 ± 1.0	4.7 ± 1.6	3.5 ± 1.1	4.8 ± 0.7
Stomach	1.0 ± 0.3	1.0 ± 0.3	0.8 ± 0.3	0.9 ± 0.2
Small Intestine	1.8 ± 0.2	1.4 ± 0.4	1.1 ± 0.3	1.2 ± 0.2
Large Intestine	1.2 ± 0.1	0.9 ± 0.3	0.7 ± 0.2	0.8 ± 0.2
Kidneys	6.7 ± 2.9	4.6 ± 1.1	4.9 ± 1.1	4.4 ± 0.3
Muscle	1.3 ± 0.2	0.9 ± 0.2	0.9 ± 0.3	0.8 ± 0.1
Bone	2.4 ± 0.4	3.7 ± 0.9	3.3 ± 0.7	5.0 ± 1.5

Table S3. Tumor-to-background activity concentration ratios calculated using the biodistribution data from athymic nude mice (n = 5 per time point) bearing SKOV3 human ovarian cancer xenografts collected 24, 72, and 120 h after the intravenous administration of $[^{89}Zr]Zr$-DFO-AR20.5 (0.65 – 0.69 MBq; 17.6 – 18.6 μCi; 6.6 – 7.0 μg). For the 72 h blocking experiment, the mice were administered the same amount of $[^{89}Zr]Zr$-DFO-AR20.5 mixed with an excess of unmodified AR20.5 (~500 μg per mouse). Stomach, small intestine, and large intestine values include contents.

**Tumor-to-**	**24 h**	**72 h**	**72 h block**	**120 h**
Blood	0.8 ± 0.3	1.9 ± 0.6	0.7 ± 0.3	3.6 ± 1.2
Heart	2.9 ± 1.2	7.1 ± 2.2	2.3 ± 1.0	12.5 ± 4.3
Lungs	1.4 ± 0.6	3.9 ± 1.6	1.3 ± 0.7	7.3 ± 2.5
Liver	1.8 ± 0.8	3.7 ± 1.0	1.2 ± 0.7	5.4 ± 2.0
Spleen	2.7 ± 1.1	4.8 ± 1.9	2.0 ± 1.0	6.9 ± 2.5
Stomach	11.5 ± 5.4	21.9 ± 8.2	8.7 ± 4.4	35.7 ± 14.1
Small Intestine	6.6 ± 2.5	15.7 ± 5.2	6.4 ± 3.0	27.7 ± 9.9
Large Intestine	10.0 ± 3.6	24.8 ± 9.1	9.8 ± 4.4	43.6 ± 18.3
Kidneys	1.8 ± 1.0	4.9 ± 1.6	1.7 ± 0.8	7.5 ± 2.6
Muscle	9.3 ± 3.5	24.6 ± 8.0	7.4 ± 3.5	42.7 ± 14.6
Bone	4.8 ± 1.8	6.1 ± 2.0	2.1 ± 0.9	6.6 ± 3.0

Table S4. Biodistribution data from athymic nude mice (n = 3) bearing SKOV3-Red-FLuc human ovarian cancer orthotopic xenografts collected 120 h after the intravenous administration of [^{89}Zr]Zr-DFO-AR20.5 (5.6 – 6.0 MBq; 150.6 – 161.4 μCi; 59.1 – 63.3 μg) or [^{89}Zr]Zr-DFO-mIgG (6.1 – 6.4 MBq; 163.7 – 171.7 μCi; 61.5 – 70.1 μg) via the tail vein. The values are %ID/g ± SD, and the stomach, small intestine, and large intestine values include contents.

**Tissue**	**[^{89}Zr]Zr-DFO-AR20.5**	**[^{89}Zr]Zr-DFO-mIgG**
Blood	2.4 ± 2.1	4.2 ± 1.1
Tumor	11.3 ± 7.1	3.1 ± 0.8
Heart	0.9 ± 0.7	1.2 ± 0.2
Lungs	1.5 ± 1.4	1.9 ±0.6
Liver	10.5 ± 2.4	5.1 ± 0.1
Spleen	6.1 ± 0.3	4.5 ± 1.6
Stomach	0.3 ± 0.1	0.3 ± 0.1
Small Intestine	0.4 ± 0.1	0.5 ± 0.2
Large Intestine	0.4 ± 0.3	0.5 ± 0.1
Kidneys	2.1 ± 0.4	5.2 ± 0.5
Muscle	0.5 ± 0.2	0.6 ± 0.2
Bone	1.7 ± 0.2	1.8 ± 0.4

Table S5. Tumor-to-organ activity concentration ratios derived from the biodistribution data from athymic nude mice (n = 3) bearing SKOV3-Red-FLuc human ovarian cancer orthotopic xenografts collected 120 h after the intravenous administration of [^{89}Zr]Zr-DFO-AR20.5 (5.6 – 6.0 MBq; 150.6 – 161.4 µCi; 59.1 – 63.3 µg) or [^{89}Zr]Zr-DFO-mIgG (6.1 – 6.4 MBq; 163.7 – 171.7 µCi; 61.5 – 70.1 µg) via the tail vein. The stomach, small intestine, and large intestine values include contents.

**Tumor to-**	**[^{89}Zr]Zr-DFO-AR20.5**	**[^{89}Zr]Zr-DFO-mIgG**
Blood	4.6 ± 4.9	0.7 ± 0.3
Heart	12.9 ± 12.6	2.5 ± 0.8
Lungs	7.4 ± 8.0	1.6 ± 0.6
Liver	1.1 ± 0.7	0.6 ± 0.2
Spleen	1.9 ± 1.2	0.7 ± 0.3
Stomach	33.2 ± 23.5	11.9 ± 5.1
Small Intestine	30.4 ± 19.7	6.5 ± 2.8
Large Intestine	27.5 ± 25.9	6.8 ± 2.4
Kidneys	5.3 ± 3.5	0.6 ± 0.2
Muscle	24.6 ± 19.3	5.1 ± 2.2
Bone	6.6 ± 4.2	1.8 ± 0.6

Table S6. Biodistribution data for the metastatic lesions collected from athymic nude mice (n = 3) bearing SKOV3-Red-FLuc human ovarian cancer orthotopic xenografts 120 h after the intravenous administration of [^{89}Zr]Zr-DFO-AR20.5 (5.6 – 6.0 MBq; 150.6 – 161.4 μCi; 59.1 – 63.3 μg) via the tail vein. The values are %ID/g.

Mouse	Metastatic Lesions	[^{89}Zr]Zr-DFO-AR20.5
Mouse 1	*Abdominal*	9.3
	Peritoneal 1	6.5
	Peritoneal 2	7.4
Mouse 2	*Peritoneal*	29.0
Mouse 3	*Peritoneal*	5.2
	Hepatic	23.0
	Right Ovarian 1	13.5
	Right Ovarian 2	5.5
